# Gender scores in epidemiological research: methods, advantages and implications

**DOI:** 10.1016/j.lanepe.2024.100962

**Published:** 2024-06-14

**Authors:** Aranka V. Ballering, Tim C. Olde Hartman, Judith G.M. Rosmalen

**Affiliations:** a University of Groningen, University Medical Center Groningen, Department of Psychiatry, P.O. Box 30.001, 9700 RB, Groningen, the Netherlands; b Department of Primary and Community Care, Radboud Institute of Medical Innovation, Radboud University Medical Center, P.O. Box 9101, 6500 HB, Nijmegen, the Netherlands; c University of Groningen, University Medical Center Groningen, Department of Internal Medicine, P.O. Box 30.001, 9700 RB, Groningen, the Netherlands

**Keywords:** Gender-sensitive healthcare, Sex-sensitive healthcare, Epidemiology

## Abstract

Sex and gender-related factors are strongly associated with patients' illness trajectories, underscoring their essential role in epidemiological research and healthcare. Ignoring sex and gender in research and health inevitably results in inequities between women and men in terms of detection of disease, preventative measures, and effectiveness of treatment. Historical influences, including ideas of female inferiority and conservative notions of women's health only comprising reproductive health, reinforced the perceived irrelevance of sex and gender to health. Currently, these ideas are largely abandoned and epidemiology is becoming increasingly sensitive to sex. Gender-sensitivity, however, is lagging behind. This is potentially due to lacking knowledge and awareness about the relevance of both sex and gender to health and challenges in operationalizing gender in epidemiological research. Here, we thoroughly discuss the relevance of sex and gender to health, and pay special attention to the time, place, and culture-dependent embodiment of gender. We also discuss the operationalization of gender via composite gender scores in epidemiological studies. We argue to move beyond solely using these. Rather we should consider sex and gender in the initial stages of designing a study, to facilitate relevant, reproducible, and person-centric research.

## Introduction

Sex and gender-related factors are present in many, if not all medical disciplines.^1^ The effects of sex and gender of patients are seen across symptoms,^2^ diseases,^1^ and throughout all phases of the patients’ illness trajectories. Differences between women and men are identified in the biopsychosocial etiology and persistence of somatic symptoms,^3^, ^4^, ^5^ help-seeking behaviors,^6^ medical communication about symptoms,^7^ provided diagnostic interventions,^8^ and efficacy of treatment.^9^

A lack of knowledge on biological and psychosocial factors pertaining to women and men hinders early detection of disease, better primary and secondary preventative measures, and more effective and personalized treatments.^10^ This lack of knowledge has been reinforced by historical influences, including ideas of female inferiority and conservative notions of women's health being strongly intertwined with merely reproductive health. Currently, these notions are largely abandoned and researchers and healthcare professionals aim for inclusive healthcare for all sexes and genders.^11^ The effects of sex and gender-related factors in health gain worldwide attention in policymaking, clinical practice, and health-related research. For example, the Sex And Gender Equity in Research (SAGER) guidelines are now widely adopted in editorial policies,^11^^,^^12^ encouraging a clear operationalization of sex and gender, and sex and gender-stratified reporting of research results if appropriate.

Although epidemiological research has been becoming increasingly sex-sensitive (i.e., attentive towards the biology of female and male bodies, and bodies with intersex variations), gender-sensitivity (i.e., attentive towards societal norms, pressures, and mores related to being a man, woman, or other identity) remains relatively scarce. The few available epidemiological studies that include a gender measure apply a methodology that is not robust.^13^^,^^14^ A lack of gender-sensitivity in epidemiology, and health-related research in general, is problematic as we know that many healthcare decisions are affected by the socially-prescribed norms and experiences of patients ‘being a man’ or ‘being a woman’.^15^ Additionally, disregarding gender in health and research hampers personalized medicine, but also rigor, validity, and generalizability of the scientific process.^16^ Multiple reasons could underlie this lack of gender-sensitivity in epidemiological research. First, it could be due to lacking knowledge or awareness about gender among researchers, stemming from the continuous misconception that gender conflates with sex. Second, the epidemiological operationalization of gender is very challenging, since it refers to a socially-constructed concept encompassing multiple dimensions.

How people shape gender is a dynamic process, and highly dependent on place, time, and society. This complicates any generalization of gender over epidemiological studies. Since little direct and consistent information on any aspect of participants’ gender is traditionally collected in epidemiological studies,^17^ an increasingly popular strategy to operationalize gender is via composite gender scores or indices that approach (dimensions of) gender using previously-collected psychosocial variables. These scores have their own specific caveats. In this paper we reflect upon the recent trend of operationalizing gender in epidemiological research via composite scores. We argue that these scores allow us to obtain initial knowledge on the role of gender in health, but that we should ultimately move beyond these. First, we will provide a concise explanation of sex and gender. Second, we will systematically describe composite gender indices and discuss the concomitant advantages and disadvantages in underlying methodology. Third, we will propose a way forward to achieve sex and gender-sensitivity in epidemiological research.

## Sex, gender, and health

A prominent misconception in the debates regarding sex and gender sensitive medicine revolves around the conflation of sex and gender. Recent research shows that 29% of the scientific biomedical articles claiming to assess gender differences actually focus on sex differences.^18^ This blurring of the concepts mixes up the relative contribution of sex and gender to health outcomes, ultimately affecting the validity of conclusions drawn from research and thereby the efficacy of policymaking and health interventions. Although sex and gender are continuously shaping each other, the two concepts are different and we do not necessarily consider one as the direct consequence of the other.

On the one hand, sex encompasses the biology of bodies. It refers to the biological features and aspects, such as physiology, anatomy, gene expression, and hormone levels and function that define female and male bodies.^17^ Sex is usually assigned at birth. It is not a dichotomy. Although it is often considered as such, it is rather a continuum ranging from male to female and vice versa. Intersex variations occur within bodies as well: bodies with intersex variations do not conform to the archetypical medical and cultural ideas of what constitutes a male or female body.^2^

**Fig. 1:**
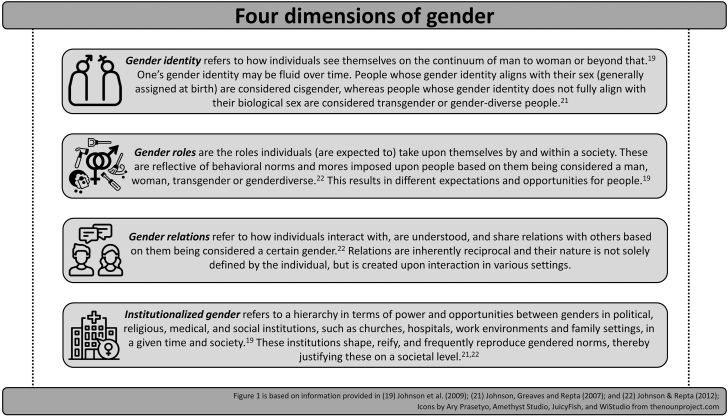
The four dimensions of gender.

Gender, on the other hand, refers to a dynamic, multidimensional, and socioculturally constructed concept, which is strongly dependent on society. This means that the embodiment of gender may differ between geographical regions, time, and cultures. Although gender is frequently regarded as the psychosocial equivalent of sex, no direct causality should be conferred from that notion as it may unintentionally reinforce the idea that gender is a sole consequence of sex. Gender encompasses the dynamic embodiment of identities, behaviors and roles within a given society.^19^ An increasing body of evidence shows that multiple symptoms, diseases, and healthcare decisions are affected by (the degree of adherence to) socially prescribed norms for, and experiences of, women and men.^15^^,^^20^ In Fig. 1 we distinguish between four dimensions of gender that we consider most relevant for epidemiological and healthcare-related research, in line with the Canadian Women's Health Research Network.^19^^,^^21^

Notably, gender has an inherent interactive aspect to it with its embodiment being subject to ever-changing societal norms. Gender consciously or unconsciously plays a role in all social interactions, relationships, and institutions, allowing for reproduction and potential reinforcement of existing norms and mores regarding hierarchies between genders.^22^ The proneness of gender to hierarchical pressures exemplifies a mechanism via which gender may intersect with other social determinants, such as socioeconomic status and class, affecting health outcomes.^23^ This indicates that gender is seldom a stand-alone factor when affecting patients’ illness trajectories, but rather should be considered in its context of other social factors.

## Operationalization of gender via gender indices

Traditionally gender did not have a prominent place in epidemiological studies. It was considered to be a mere consequence of, and therefore similar to, people's sex assigned at birth.^17^ Only by the 2000's the scientific movement that aimed to incorporate gender next to sex in epidemiology and health-related research gained significant momentum, as sex and gender sensitivity became anchored in research policies.^16^ Research since then has shown that sex and gender have distinct influences on health, operating via different pathways, but do interact when affecting health outcomes.^1^^,^^2^^,^^24^ Since many large-scale cohort studies were already initiated before sex and gender sensitivity became solidified in research policies, these typically did not include intentional measures to gather information on any aspect of participants' gender. Over time, this stimulated the development of comprehensive secondary methods to assess gender in epidemiological studies that lacked direct gender measures in the initial data collection. Especially composite gender indices have gained increased attention as a method to assess gender in cohort studies when information hereon is lacking.^25^ In composite gender indices, the culture-specific adherence to gendered factors that associate with being of female or male sex is determined. It is a method to capture (dimensions of) gender in numbers. Gender indices are developed in newly initiated cohort studies as well. These are considered advantageous as these do not require additional items to assess individual's gender. This avoids supposedly sensitive questions and increased participant burden, and thus participant drop-out.

### Search strategy and selection criteria

To obtain a systematic overview of secondary, composite gender indices developed in existing cohort studies and the underlying methods, we searched PubMed, Web of Science, and CINAHL up until 1 February 2024 using the search terms as described in Appendix 1. The search yielded 877 articles and we included 4 additional articles based on expert opinion. After exclusion of 351 duplicates, 530 abstracts were screened.^26^ We excluded 487 articles, and screened the full text of 41 articles. Ultimately, 24 articles that describe 26 gender indices are included in this review (Table 1, Appendix 2).^2^^,^^27^, ^28^, ^29^, ^30^, ^31^, ^32^, ^33^, ^34^, ^35^, ^36^, ^37^, ^38^, ^39^, ^40^, ^41^, ^42^, ^43^, ^44^, ^45^, ^46^, ^47^, ^48^, ^49^

**Table 1:** Overview of the included studies, their characteristics, and the respective composite gender indices.

	Authors (year)	Methodology		Cohort information	Gender measure	Included components in composite gender indices
		Theory-driven	Data-driven			
1	Van den Houdt et al. (2024)^27^	x		THORESCI-Gender study (The Netherlands; 2020–2021; N = 532; 16% female)	Gender norm score, ranging from 0 to 10 with higher scores indicating female gender roles	(1) Civil status (2) Occupational status (3) Primary earnership (4) Education level (5) Household task division
2	Vader et al. (2023)^28^	x		Doetinchem Cohort Study (The Netherlands; 2008–2012; N = 4017; 53% female)	Masculine gender score, ranging from 0 to 19 with higher scores referring to more masculine connotated aspects of everyday life	(1) Work and education a Division of paid work between respondent and partner b Physical intensity of work c Educational level compared to partner (2) Informal care a Time spent on household chores b Time spent on odd jobs c Frequency of taking care of sick people (3) Lifestyle a Physical intensity/type of sport b Smoking cigars or pipe c Type of alcohol consumption (4) Emotions a Limitations in work and activities due to emotional problems b Experiencing feelings of nervousness c Feeling energetic and vibrant d Feeling exhausted and tired
3	Mommersteeg et al. (2023)^29^	x		Duch community sample (The Netherlands; 2019; N = 678; 54% female)	Gender norm score, ranging from 0 to 14 with higher scores indicating a feminine profile	(1) Civil status (2) Education level (3) Employment status (4) Primary earnership (5) Household responsibilities (6) Caretaking of children at home (7) Informal caregiving
4	De Breij et al. (2022)^30^	x		Longitudinal Aging Study Amsterdam (The Netherlands; 2012–2013 and 2015–2016; N = 313; 43% female)	Gender index, ranging from 0 to 22, dichotomized with >7 indicating a feminine index	(1) Working hours (2) Income (3) Occupation segregation (4) Education (5) Informal caregiving (6) Time spent on household tasks
5	Smith and Koehoorn (2016)^31^	x		Canadian Labour Force Survey (Canada; 1997; N = 696,350; 2014; N = 729,132; % female not reported)	Labour Force Gender Index (LFGI), ranging from 0 to 10 with lower scores indicating masculine labor market gender roles	(1) Responsibility for caring for children a Level of reduction in labor market participation due to family responsibilities (2) Occupational segregation a Male-dominated occupation (3) Hours of work relative to partner/spouse (4) Education relative to partner/spouse
6^a^	Lacasse et al. (2020)^32^	x	x	Canadian Community Health Survey (Canada; 2007–2012; N = 29,470; 47% female)	GENDER index, ranging from 0 to 100 with higher scores indicating having more feminine characteristics	(1) Occupation and education (2) Household composition and income (3) Racial/cultural group (4) Ownership of the household (5) Sense of belonging to the local community (6) Frequency of experienced stress
7	Levinsson et al. (2022)^33^	x	x	UK Biobank (United Kingdom, 2006–2010; N = 315,937; 53% female)	Femininity Score, standardized in the general population, expressed in standard deviations	(1) Education (2) Occupational status (3) Depression (4) Risk taking (5) Neuroticism (6) Birthyear
8	Nauman et al. (2021)^34^	x	x	Berlin Aging Study II (Germany, 2009–2014; N = 1869; 51% female)	Gender score, ranging from 0 to 100 with higher scores indicating having more feminine characteristics	(1) Chronic stress (2) Marital status (3) Risk taking behavior (4) Agreeableness (5) Neuroticism (6) Extraversion (7) Loneliness (8) Conscientiousness (9) Educational level
9	Pelletier et al. (2015)^35^	x	x	GENESIS-PRAXY (Canada, USA, and Switzerland; 2009–2013; N = 1075; 32% female)	Gender score, ranging from 0 to 100 with higher scores indicating having more feminine gender-related characteristics	(1) Primary earner status (2) Personal income (3) Number of hours per week doing housework (4) Primary responsibility for doing housework (5) Level of stress at home (6) Bem Sex Role Inventory—masculinity score (7) Bem Sex Role Inventory—femininity score
10	Azizi et al. (2021)^36^	x	x	Canadian Community Health Survey (Canada; 2014; N = 63,522; 55% female)	Gender score, ranging from 0 to 1 with higher scores indicating characteristics traditionally ascribed to women	(1) Household size (2) Education (3) Perceived life stress (4) Sense of belonging to community (5) Marital status (6) Household income
11	Azizi et al. (2021)^36^	x	x	Austria Health Interview Survey (Austria; 2014; N = 15,671; 56% female)	Gender score, ranging from 0 to 1 with higher scores indicating characteristics traditionally ascribed to women	(1) Education (2) Household income (3) Marital status (4) Frequency of negative emotions
12	Gisinger et al. (2023)^37^	x	x	Canadian Community Health Survey (Canada; 2015–2016; N = 109,659; 54% female)	Gender score, ranging from 0 to 1 with higher scores representing characteristics traditionally ascribed to women	(1) Household size (2) Education level (3) Perceived life stress (4) Sense of belonging to community (5) Marital status (6) Household income
13	Gisinger et al. (2023)^37^	x	x	European Health Interview Survey (EU member states, Iceland, and Norway, 2013–2015; N = 316,333; 51% female)	Gender score, ranging from 0 to 1 with higher scores representing characteristics traditionally ascribed to women	(1) Household size (2) Education level (3) Marital status (4) Household income
14	Yuan et al. (2021)^38^	x	x	Health and Retirement Study cohort (USA, 2016; N = 2912; 51% female)	Gender score, ranging from 0 to 100, with higher scores indicating femininity	(1) Willingness to take risks (2) Loneliness (3) Participation in household tasks (4) Alcohol intake (5) Depression
15^b^	Pohrt et al. (2023)^39^	x	x	Gend Age Study (Germany; 2018–2020; N = 1100; 52% female)	Gender score, ranging from 0 to 1, with higher values indicating femininity scores	(1) Education and income (2) Household and care responsibilities and time spent hereon (3) Social support (4) Civil status (5) Gender-related personality traits (6) Bem Sex Role Inventory – masculinity score (7) Bem Sex Role Inventory – femininity score (8) Perceived social standing in community and Germany
16	Mena et al. (2023)^40^	x	x	German Socio-Economic Panel (Germany; 2017; N = 23,159; 54% female)	IG-Score, ranging from 0 to 1 with higher values indicating femininity	(1) Age (2) Employment status (3) Occupational status (4) Education (5) Partner in household (6) Children <16 years in household (7) Migration background (8) Household language (9) Feeling lonely (10) Disability status (11) Urbanity (12) Household help
17^c^	Wandschneider et al. (2022A)^41^	x	x	German Socio-Economic Panel (Germany; 2018; N = 20,767; 57% female)	Gender score, ranging from 0 to 1, with higher values indicating femininity	(1) Attitudes and norms towards gender roles (2) Economic and power relations (3) Affective relations/emotional resources
18^d^	Wandschneider et al. (2022B)^42^	x	x	German Socio-Economic Panel (Germany; 2018; N = 20,897; 56% female)	Gender score, ranging from 0 to 1, with higher values indicating femininity	(1) Attitudes and norms towards gender roles (2) Economic and power relations (3) Affective relations/emotional resources
19^e^	Fleming et al. (2017)^43^	x	x	National Longitudinal Study of Adolescent to Adult Health—4th wave (USA; 2008–2009; N = 9417; 55% female)	Measurement technique for assessment of performing gender, ranging from 0 to 1 with higher scores indicating being male	(1) Lifestyle (2) Arrested by police (3) Personality traits and intelligence (4) Type of leisure and sports activities (5) Mental Health (6) Engaging in risky behaviors (7) Perceived stress, worries and tiredness (8) Sympathy for and interest in others
20	Cipriani et al. (2024)^44^		x	Signature Biobank (Canada; 2012–2020; N = 1708; 39% female)	Gender index score, ranging from 0 to 1, with higher values indicating masculinity	(1) Hostile behavior during childhood and adulthood (2) Having at least a secondary school diploma or bachelor's degree (3) Sleep satisfaction and efficacity (4) Having private housing (5) Experiences of sexual violence during childhood
21^f^	Raparelli et al. (2023)^45^		x	EVA Cohort (Italy; 2016–2020; N = 311; 38% female)	EVA gender score, ranging from 0 to 100, with higher scores relating to characteristics traditionally ascribed to women.	(1) Engagement in social leisure activities (2) Marital status (3) Responsible for household tasks (4) Time spend on household tasks (5) Primary earnership (6) Level of stress at home (7) Level of received emotional support (8) Availability of trust and confidence measures
22^g^	Teterina et al. (2023)^46^		x	Health administrative data for publicly funded services, Ontario (Canada; 2002–2020; N = 276,812; 45% female)	Gender score, ranging from 0 to 1, with higher values indicating ‘woman-like’ characteristics.	(1) ICD-10 CA diagnostic codes
23^h^	Ballering et al. (2020)^2^		x	Dutch Lifelines Cohort Study (The Netherlands, 2006–2014; N = 152,728; 59% female)	Gender index, ranging from 0 to 100 with higher scores indicating having more feminine characteristics	(1) Type of leisure activities (2) Occupation-related components (3) Time spend on household tasks (4) Time spend on odd jobs (5) Lifestyle (6) Experiencing long-term difficulties or negative life events (7) Personality traits and emotions
24	Lippa (1995)^47^	No explicit theoretical considerations for variable selection reported	x	Psychology students (USA, Period of data collection not reported; N = 227; 48% female)	Composite measure of gender-related behaviors, no range provided	(1) SAT – math scores (2) Mental rotation scores (3) Rating of masculinity or femininity in self-describing paragraphs (4) Rating of masculinity or femininity in college majors (5) Self-reported aggressiveness (6) Smiling as coded from photographs (7) Appearance (8) Handwriting styles
25	Lippa and Connely (1990)^48^	No explicit theoretical considerations for variable selection reported	x	Psychology students (USA, Period of data collection not reported; N = 227; 48% female)	Gender diagnosticity measure, ranging from 0 to 100 with higher scores indicating more masculine characteristics	(1) Occupational preference
26	Reany and Ferguson (1974)^49^	No explicit theoretical considerations for variable selection reported	x	University students (USA, Period of data collection not reported; N = 2415; 66% female)	M-F scale, ranging from −99±74 with higher scores indicating more feminine adjectives	(1) Cold-warm adjective scale consisting of 80 item-pairs

a As 19 components (43 dummy variables) were included in the GENDER Index, we summarized these for reasons of clarity. A full overview of the included components is provided in the original study.

b As 15 components were included in the Gender score, we summarized these for reasons of clarity. A full overview of the included components in provided in the original study.

c The study's authors summarize the 13 included variables in the Gender score under these headings. A full overview of the included components is provided in the original study.

d The study's authors summarize 14 and 15 included variables in the Gender score under these headings for an immigrant and non-immigrant sample in Germany, respectively. A full overview of the included components is provided in the original study.

e The measurement technique for assessment of performing gender included 22 items at the 4th wave. Therefore, we summarized these for reasons of clarity.

f The authors note that they initiated the development of the EVA gender score with all 45 gender-related variables that were assessed in the EVA cohort, but do not explicitly describe which considerations underlie the selection of these 45 variables. As all variables were included, we consider this a data-driven gender score.

g 281 ICD-10-CA diagnostic codes were included to develop the gender score. A full overview of the included ICD-10-CA diagnostic codes can be found in the original study.

h As 153 (dummy) variables that represented 85 unique psychosocial variables were included in the Gender index, we summarized these for reasons of clarity. A full overview of the included components is provided in the original study.

Inclusion of articles was based on the following set of criteria: (1) Studies had to develop a secondary, composite gender measure based on variables available in cohorts; (2) the developed gender measure had to be applicable to the individual adult participants in the cohort (e.g., no parent-reported measures for children or measures on country level); (3) the full text of studies had to be available in Dutch or English.

### Description of composite gender indices

We identified 24 studies that developed 26 composite gender indices in cohorts ranging from 227 to 729,132 individuals. All included gender indices are developed in Europe or North America. This may be one of the reasons why the components included in the gender indices overlap across multiple studies. Components related to occupation, income, education, civil status, care-giving and household responsibilities, and ways of spending (leisure) time are found in most of the gender indices, irrespective of the method used to construct the gender index. The majority of the indices are developed by combining elements from theory-driven and data-driven approaches. These approaches will be discussed more extensively below. The recent interest in gender-sensitivity in epidemiology is illustrated by the fact that most composite gender indices are developed from 2015 onwards. It should be noted that some indices are developed in patient cohorts, for example among patients with cardiovascular disease or in a subgroup of the general population that experienced traumatic brain injury.^35^^,^^45^^,^^46^ Components of developed indices in these populations may therefore be disease-specific.

## Methods underlying composite gender indices

Recently, researchers have questioned methodologies underlying composite gender indices and whether these should be theory-driven or data-driven.^25^^,^^50^ In theory-driven indices, (psychosocial) variables that provide information on participants' gender are selected based on theory or expert opinion and subsequently combined into one composite score. Data-driven indices are generally developed based on the premise that the distribution of psychosocial factors differs between the sexes in a given population. The absence or presence of gendered factors are then measured in an individual, relative to the distribution of these gendered characteristics in the populations' male and female participants (i.e., the gender diagnosticity approach).^48^^,^^50^ In other words, it assesses the extent to which an individual complies with study-specific feminine or masculine factors. Practically it involves the selection of (psychosocial) variables that optimally predict the participants' sex by an algorithm, that simultaneously indicates the extent to which the psychosocial variable represents feminine or masculine characteristics. The total weighted score of these predicting variables determines one's level of adherence to gendered factors. Theory-driven and data-driven methodologies are frequently combined as well. For example, gender-related variables are selected based on theory or expert opinion and subsequently included in principal component analyses to identify gender-related principal components and to reduce the number of variables. Thereafter, the association between retained variables and participants' sex is assessed with non-significant variables being excluded. The significant associations are condensed into a propensity score that indicates the level of adherence to feminine or masculine factors. Below we discuss the advantages and disadvantages of the methods underlying composite gender indices. It should be noted that gender indices are not reflective of participants' gender identity. This dimension of gender is to be assessed by direct items that allow participants to at least indicate their sex assigned at birth and current gender identity.^51^

### Theory-driven composite gender indices

Theory-driven gender indices have been increasingly applied recently (Table 1), but may be limited in their utility for a variety of reasons. First, some of the developed gender indices do not take into account the broadness and multidimensionality of gender, but merely focus on one domain. An example is the Labor Force Gender Index that focusses solely on people's gender roles and institutionalized gender in relation to their occupation, disregarding other important factors such as leisure activities, lifestyle and (social) mobility that may be a part of the embodiment of gender.^31^ Second, once developed, the contents of a theory-driven gender index are static, while the embodiment of gender is a dynamic process, dependent on time, place and culture. Therefore, a constant redefinition of the gendered components included in a theory-driven gender index is required. For example, the recently developed one-dimensional masculine gender score in the Dutch Doetinchem Cohort Study includes variables on education, with an educational level higher than one's partner indicating masculinity.^28^ In the Netherlands the proportion of women with a high educational level has been steadily increasing, surpassing that of men in the early 2000's.^52^ This shows how gendered components evolve over time, but also that certain components of a gender index may be considered masculine in one subgroup (e.g., older generations), and feminine in other subgroups (e.g., younger generations). Third, theory-driven gender indices rely heavily on expert knowledge. However, experts have their own frame of reference and are not free from bias, potentially reinforcing sexism or other biases in the development of gender indices. Fourth, the components of fully theory-driven gender indices frequently have an equal weight in defining femininity and/or masculinity, while these may differ in their extent of contributing to femininity and masculinity. This results in imprecise operationalizations of gender.

### Data-driven composite gender indices

Despite the limitations of theory-driven gender indices, a fully data-driven gender index is not automatically preferable in all study designs. In data-driven indices an algorithm assesses whether and how strongly psychosocial variables are associated to male or female sex, usually based on the gender diagnosticity approach. This method is versatile, flexible, and applicable to many studies, as it accounts for the time, place, and culture-sensitivity of gender. Yet, such a gender index is defined as a unidimensional, bipolar scale.^2^ Herein the underlying algorithm identifies psychosocial components that either predict female or male sex, reinforcing mutual exclusivity. Subsequently, participants' individual scores on the gender index vary within a continuum of feminine or masculine scores. A middle score indicates androgyny, referring to a balance in weighted masculine and feminine psychosocial variables.^2^ As psychosocial variables that do not associate significantly with male or female sex are excluded by the underlying algorithm, gender indices developed by these methods cannot indicate a lack of both feminine and masculine characteristics. It is difficult to counter this, as generally data-driven gender indices are derived from a form of logistic regression analyses. Herein the outcome (i.e., participants’ sex) is dichotomous, with the inverse of male sex automatically being female sex. Another aspect to consider when calculating a data-driven gender index per participant via a machine learning method is that large datasets are required that allow for sufficient variance of the included predictors.^2^ The availability of many variables in a cohort allows for testing multiple combinations of factors that most optimally associate with female or male sex. This is advantageous for the validity of the analyses. The included data should be of high quality, as a data-driven gender index is only as good as the dataset on which the algorithm is trained.

The conceptual underpinnings of data-driven gender indices have been questioned.^25^^,^^50^ In gender indices with a data-driven component, sex is regressed on a large variety of psychosocial variables that aim to capture gender. This relies on the fundamental notion of gendered characteristics being derived from sex assigned at birth. This also touches upon one of the main critiques towards data-driven gender indices: a potentially unjustified belief that a data-driven gender index is fully independent from sex.^25^^,^^50^ Multiple indications exist that support sex and gender indices’ independence in cohorts. First, assessments of multicollinearity confirm that statistical models are able to sufficiently disentangle sex and gender indices, indicating two statistically separate entities. A second indication of the independence of sex and data-driven gender indices is the great variability in gender scores in both female and male participants.^2^^,^^28^^,^^32^^,^^33^^,^^48^

Notably, one's ideas regarding the relationship between sex and gender influence the perceived appropriateness of defining a data-driven gender index. If one argues sex and gender to be in a continuous dialogue it is implied that a fully data-driven gender index in which sex is predicted by psychosocial variables is inadequate to capture gender since their association is simultaneous and they are too strongly intertwined. This may render the disentanglement of sex and gender in epidemiology a mere statistical and potentially clinically irrelevant exercise.

## Desirability of gender indices in research

In the end, the debate that aims to define the superior end of the methodological spectrum of gender indices is merely theoretical, as in practice nearly similar gendered components are retained in theory-driven and data-driven indices.^2^^,^^28^^,^^31^, ^32^, ^33^, ^34^, ^35^ It is especially worth mentioning that the Dutch indices, which are defined via different methods,^2^^,^^27^, ^28^, ^29^, ^30^ include highly similar components to define femininity and masculinity.

However, debating the most adequate one-size-fits-all methodology for gender indices does not align with the context-dependent nature of gender.^42^ Rather, the applied methodology should be compatible with one's ideas regarding the relationship between sex and gender, study design, research question, and sample size. For example, applying a data-driven approach to gender, allows for a cross-cultural comparison of the gendered components that are included in gender indices across cultures and societies. In contrast, a small sample size or research question that focusses on a specific gender dimension could call for a theory-driven gender index.

### Desirability of gender indices in epidemiology

The question remains whether gender indices are the most adequate and valid gender measure in epidemiological research.^50^ Gender indices are useful and pragmatic tools to obtain a gender measure if no information hereon is collected, but should not be treated as an absolute truth. Components on which gender indices are based may not be bias-free.^25^ An inherent gender bias could occur in the construction of survey items or in data collection.^53^ The former is exemplified by survey items that reproduce conservative ideas regarding the distribution of power between men and women, implying superiority of one gender over another.^53^ For example, agreeableness with the statement ‘If a woman earns more money than her husband, it's almost certain to cause problems' or ‘When a mother works for pay, the children suffer’ is assessed in seventh wave of the large-scale World Value Survey that collected data between 2017 and 2021 from 64 countries (N = 94,278), including the Netherlands.^54^ A similar self-perpetuating gender-bias in data collection is illustrated by survey items that assess domains that are stereotypically considered as explicitly female (e.g., children's health) or male (e.g., tobacco use) and are therefore not assessed in the opposite sex.^53^ These biases are bound to affect potential components included in gender indices. Furthermore, although specific components in a gender index may strongly associate with health outcomes, these may be obscured when combined with other gendered components into a comprehensive gender index.^24^

## How to move forward?

In clinical practice especially data-driven gender indices may prove useful as an additional tool to personalize healthcare. As gender indices are able to identify gendered factors that differentiate between men and women in a subpopulation these could inform person-centric treatment decisions and patient care. For example, the gendered factors may guide healthcare providers when taking male and female patient's medical history. Gendered factors may also prove to be a point of departure when discussing, among others, lifestyle advice and support with male and female patients.

Similarly, in epidemiological studies gender indices are informative. However, these should be the bare minimum when it comes to gender sensitivity in research. Preferably, the epidemiological community should move beyond solely using gender scores. Gender scores are frequently an indication of lacking gender considerations in the design of a study. Rather than combining multiple components into one score or index, which is subsequently interpreted as gendered, it would be more fruitful to complement these gender indices with multiple self-reported gender measures that represent different dimensions of gender in cohort studies.^17^^,^^55^

Researchers often provide two arguments against using such self-reported measures in cohort studies. First, researchers may be hesitant to include self-reported gender measures in cohorts, as the topic is supposedly too sensitive to assess and will impact the retention of participants. Second, researchers argue that it may be difficult for participants to differentiate between sex, gender, and relevant dimensions of gender in self-reported measures, which results in inconsistent data collection. A self-reported gender measure should not conflate sex and any dimension of gender, conflate gender identity with a gender diverse identity, or reduce gender to merely gender identity. Therefore, a self-reported gender measure should ideally be developed in collaboration with an inclusive and diverse participant panel to ensure acceptability among participants and compliance with local mores.^51^ It should also include a concise introduction that conceptually clarifies sex, gender and gender's relevant dimensions for participants. Recently, items that allowed for self-report of various dimensions of gender were developed in collaboration with a participant panel were introduced in the large-scale Dutch Lifelines Cohort Study (N >167.000).^17^ Herein the conventional two-step approach, which includes an assessment of sex assigned at birth and current gender identity, was complemented with two items assessing on a ten-point Likert scale the extent to which participants consider themselves masculine or feminine in terms of gender roles. A mere 0.3%–1.3% of participants did not complete this item, indicating a strong willingness among participants to share about their gender.^17^

Validity and reliability of such self-reported gender measures should be assessed structurally over time to ensure its appropriateness. This could be done by repeated cognitive interviewing with cohort participants to evaluate and potentially improve the content validity of the items. Cognitive interviewing is a qualitative method that allows for understanding the mechanisms that underly the response to a survey.^56^ Reliability could be assessed by analyzing whether results for a self-reported gender measure are reproducible in similar subsets of a cohort. Due to the dynamicity of gender, this should be an ongoing process and results of these processes cannot be directly extrapolated to cohort studies in different settings. Nevertheless, only when information on participants’ gender is directly, bias-free, and inclusively collected, we can achieve true gender sensitivity, and thus person-centric research and healthcare.

## Contributors

AB: conceptualization, data curation, investigation, methodology, project administration, visualization, writing—original draft, writing—review & editing; ToH: Funding acquisition, supervision, writing—review & editing; JR: Methodology, funding acquisition, supervision, writing—review & editing.

## Declaration of interests

JR, ToH and AB received funding from ZonMw (project number 84900013). AB received additional funding from ZonMw (project numbers 849800001 and 50018423). No further competing interests are declared.
