# Supplementary material for: Gender scores in epidemiological research: methods, advantages and implications
Source: Lancet Reg Health Eur. 2024 Jun 14;43:100962. doi: 10.1016/j.lanepe.2024.100962 (PMC11233999; doi:10.1016/j.lanepe.2024.100962)
Supplement: Supplementary Materials [file mmc1.docx]

**Appendix 1 – Search strategy**

*Pubmed (N=301)*

“gender scor*”[Text Word] OR “femininity scor*”[Text Word] OR “masculinity scor*”[Text Word] OR “gender index”[Text Word] OR “gender indices”[Text Word] OR “gender quantification”[Text Word] OR “gender scale”[Text Word] OR “femininity scale”[Text Word] OR “masculinity scale”[Text Word] OR “gender measure”[Text Word] OR “femininity measure”[Text Word] OR “masculinity measure”[Text Word] OR “secondary gender analys*”[Text Word] OR “gender norm score*”[Text Word]

*Web of Science (N=439)*

TS=(“gender scor*” OR “femininity scor*” OR “masculinity scor*” OR “gender index” OR “gender indices” OR “gender quantification” OR “gender scale” OR “femininity scale” OR “masculinity scale” OR “gender measure” OR “femininity measure” OR “masculinity measure” OR “secondary gender analys*” OR “gender norm score*”)

*CINAHL (N=137)*

TX (“gender scor*” OR “femininity scor*” OR “masculinity scor*” OR “gender index” OR “gender indices” OR “gender quantification” OR “gender scale” OR “femininity scale” OR “masculinity scale” OR “gender measure” OR “femininity measure” OR “masculinity measure” OR “secondary gender analys*” OR “gender norm score*”)

**Appendix 2 – Prisma Flowchart**

**
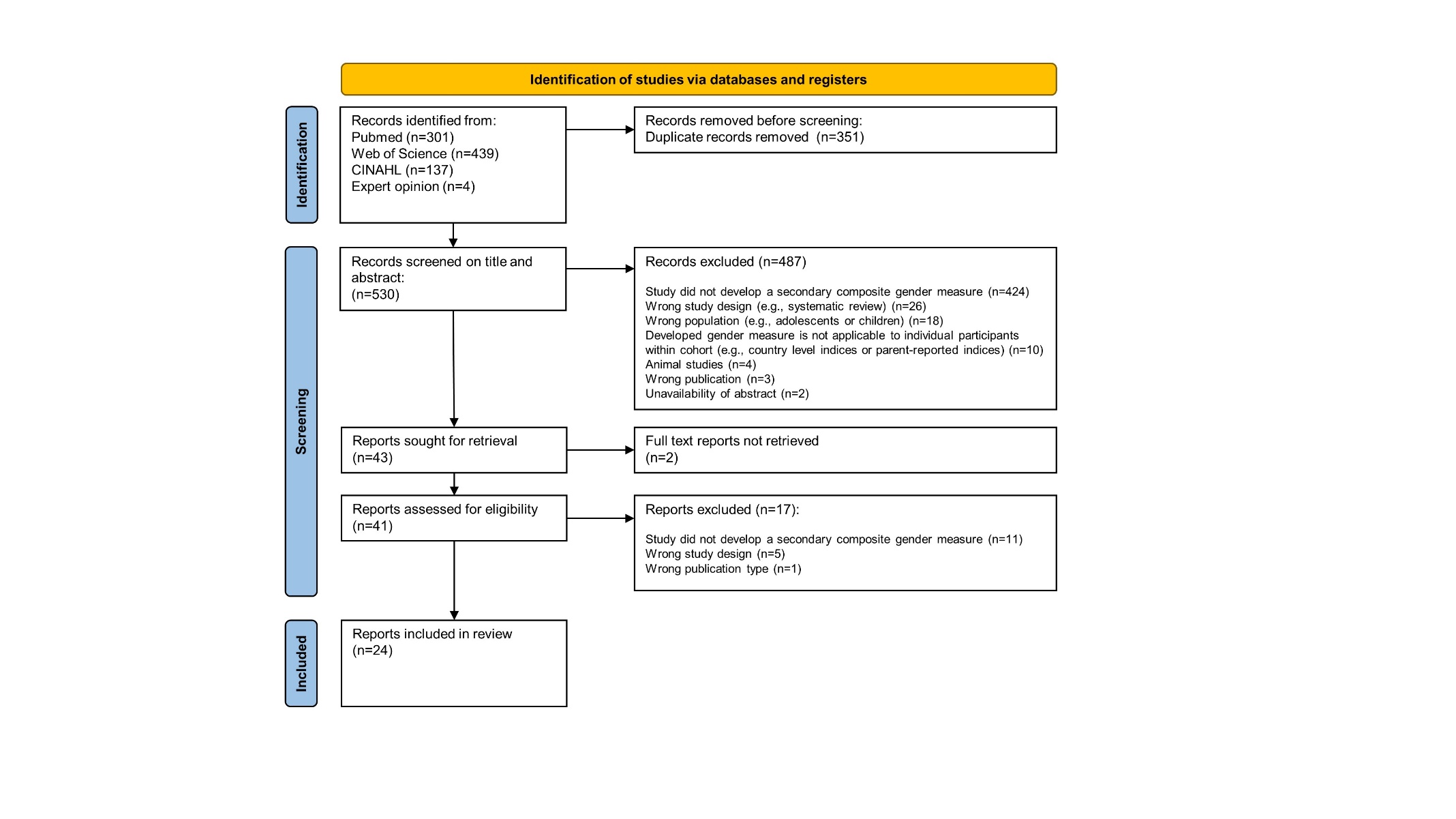
**
